# Reaction to Disease and Coping Strategies in Stressful Situations among Psoriasis Patients: Cross-Sectional Study

**DOI:** 10.3390/jcm13164693

**Published:** 2024-08-09

**Authors:** Beata Kowalewska, Marta Milewska-Buzun, Mateusz Cybulski, Andriej Szpakow, Dzmitry Khvorik, Marek Sobolewski, Piotr Aleksiejczuk, Wiaczesław Niczyporuk

**Affiliations:** 1 Department of Integrated Medical Care, Medical University of Bialystok, 15-096 Bialystok, Poland; marta.milewska-buzun@umb.edu.pl (M.M.-B.); mateusz.cybulski@umb.edu.pl (M.C.); 2 International Academy of Applied Sciences in Lomza, 18-402 Lomza, Poland; shpakofff@tut.by; 3 Department of Dermatovenerology, Medical University in Grodno, 230025 Grodno, Belarus; chvorik@mail.ru; 4 Department of Quantitative Methods, Rzeszow University of Technology, 35-959 Rzeszow, Poland; mareksobol@poczta.onet.pl; 5 Dermatology and Medical Cosmetics Clinic, 15-027 Bialystok, Poland; 6 Dermatology and Aesthetics Clinic, 15-660 Bialystok, Poland

**Keywords:** psoriasis, quality of life, disease acceptance, stress, Dermatology Life Quality Index (DLQI), Coping Inventory for Stressful Situations (CISS), Acceptance of Illness Scale (AIS)

## Abstract

**Background:** In the contemporary world, a cult of perfection is being created, and deviations from such an ideal image are becoming socially unacceptable. A particular situation arises when a defect or symptoms of a disease appear on the skin, which, in the case of people suffering from psoriasis, are a source of stress, dissatisfaction with the disease, and a reduction in quality of life. The aim of this study was to assess whether the quality of life related to the occurrence of psoriasis and the level of acceptance of the disease affect coping strategies in stressful situations. **Methods:** The study involved 111 people with common psoriasis (46.8% women and 53.2% men). Inclusion criteria were as follows: a diagnosis of common psoriasis for at least 0.5 years, no other types of psoriasis, no mental illnesses, and an informed consent of the respondent to participate in the study. In order to compile the research input, a proprietary questionnaire was used along with the following standardised tools: the Dermatology Life Quality Index (DLQI), the Acceptance of Illness Scale (AIS), and the Coping Inventory for Stressful Situations (CISS). **Results:** The duration of the disease in the studied population varied and ranged from 0.5 years to over 50 years. Most respondents showed relatively low DLQI scores, with an average value of 10.8 points. In stressful situations (CISS), the respondents primarily used a strategy based on rational thinking (Task-oriented coping), with approximately 54 points on average; followed by an avoidant style (Avoidance-oriented coping), with approximately 50 points on average; and least often an emotional style (Emotion-oriented coping), with approximately 46 points on average. The average level of disease acceptance (AIS) in the studied group equalled approximately 26 points. **Conclusions:** Psoriatic lesions on the torso caused less rational behaviour in stressful situations (a decrease in the Task-oriented coping) in women but had the opposite result in men, whereas psoriatic lesions on the head stimulated the use of Task-oriented coping in women but had the opposite result in men. The higher the acceptance of the disease (AIS) presented by the respondents, the less often they used an emotional strategy (Emotion-oriented coping) in stressful situations. The higher the quality of life (DLQI) was, the lower the values of Emotion-oriented coping were noted.

## 1. Introduction

A high level of stress or prolonged stress has a destructive impact on the entire body. It causes, among other things, a decrease in immunity, cardiovascular diseases, metabolic diseases, digestive system diseases, and fertility disorders. It may also exacerbate existing conditions or induce previously non-existent conditions [1,2]. Psoriasis is also found to be among such conditions, in which stress plays a significant role both in the appearance of the first symptoms and in the exacerbation of the disease. It should also be emphasised that psoriasis itself, along with its associated symptoms, is primarily located on the skin and thus visible to others, and is therefore a source of stress for those affected. The emotional difficulties experienced by people with psoriasis stem from alienation, a lack of understanding and acceptance, exclusion, and stigmatisation by people around them. Therefore, psoriasis is often referred to as a psychosomatic disease [3,4,5].

Psoriasis is estimated to affect 2–3% of the population in Europe and 2% of the global population, with psoriatic arthritis affecting approximately one-third of this group [6,7]. It is an inflammatory, incurable dermatosis characterised by high recurrence rates. Psoriasis manifests as well-defined, scaly lesions resulting from epidermal hyperproliferation, which do not leave scars after resolution. These lesions are recurrent and may appear in new areas of the skin or at a significantly greater intensity in previously affected areas compared to previous manifestations. It should be noted that psoriasis may also affect joints, hairy skin areas, and have a destructive impact on nails [3].

The exogenous and endogenous factors predisposing to the development of psoriasis are as follows: genetic predisposition, immunological factors, diet, certain medications, infections, mechanical injuries, psychological stress, and traumatic event experiences [4,5,8,9].

In the contemporary world, a cult of perfection is created, and deviations from such an ideal image become socially disapproved. A particular situation occurs when a defect or symptoms of a disease appear on the skin, as is the case with psoriasis. The visibility of such lesions, the assumption of their contagiousness, and the belief that they originate from poor hygiene practices result in social rejection of the affected individuals, often leading to social stigma. The person with the disease is devalued, negatively marked, and marginalised in social relationships [10,11,12,13,14,15]. The societal distancing towards dermatologically ill individuals, expressions of hostility, or feelings of disgust towards them due to their symptoms generate emotional issues, social anxiety, body image disturbances, feelings of inadequacy and inferiority, and often depression among people with psoriasis. As mentioned earlier, stress experienced by patients and intense negative emotions or traumatic experience (such as the death of a loved one or job loss) exacerbate psoriatic lesions, thereby worsening the biopsychosocial functioning of psoriasis patients and intensifying their pejorative perception through the lens of extensive and severe skin lesions and heightened emotional sensitivity [16,17,18,19,20,21]. The visible manifestation of skin symptoms in the case of psoriasis also significantly affects other aspects of these individuals’ functioning. In addition to the lack of disease acceptance or reduced quality of life, a range of somatic complications is observed, including obesity, cardiovascular diseases, depression, addictions, and sexual disorders (erectile dysfunction and libido disorders, reluctance to engage in sexual activity due to skin lesions and body image alterations, and discomfort associated with lesions located in the genital area) [20,21,22,23,24,25,26,27,28,29,30,31,32,33,34].

It should be emphasised that humans are constantly exposed to stress, and in most situations, they are unable to avoid stressors and situations that affect their emotions. Individual reactions to stress and how individuals cope with them vary, and depend on personality traits, life experiences, temperament, and adopted coping strategies. Those factors also apply to individuals who are ill, including those suffering from psoriasis.

Stress coping styles are an individual’s preferences for approaching stress and include three main trends: a task-focused style, an emotion-focused style, and an avoidance-oriented style. The task-focused style is characterized by people who, in a stressful situation, take actions to identify the stress sources and solve the problem or adapt to a new reality if a solution is not possible. The emotion-focused style refers to people who, in a stressful situation, focus on the emotions that the situation has caused them. In this style, the action strategy involves experiencing a situation that creates emotions and trying to release these emotions without taking specific actions to solve the problem. People presenting an avoidance-focused style focus avoid thinking about the problematic situation that is a source of stress, e.g., by engaging in other activities and avoiding talking about these issues [35].

It should be noted that improperly chosen coping strategies by psoriasis patients and excessive emotional reactions, often disproportionate to the situation, are factors that exacerbate symptoms of the disease, thereby reducing the quality of life and increasing the Dermatology Life Quality Index (DLQI) score related to skin discomfort. Therefore, it can be assumed that the phenomenon described above is associated with the exacerbation of skin lesions in stressful situations amongst psoriasis patients and affects the results of disease acceptance measurements, which serve as indirect indicators of the quality of life in psoriasis patients. Despite the research conducted in these areas, it is important to continue such studies due to ongoing socio-cultural changes to observe developments in the described phenomenon.

The aim of the study was to assess whether the quality of life related to psoriasis and the level of disease acceptance influence coping strategies in stressful situations. The study has assumed that individual perceptions of quality of life would depend on the level of disease acceptance and coping strategies in stressful situations, and that these phenomena would be mutually determined. It was therefore assumed that the lower the perceived quality of life by the participants, the lower the disease acceptance would be, and they would correlate more with the Emotion-oriented coping or Avoidance-oriented coping strategies, and to a lesser extent with the Task-oriented coping strategy. These phenomena would intensify with a decrease or increase in the severity of skin lesions and would be modified by socio-demographic variables (such as age) and the location of skin lesions. These phenomena have been significant for the study due to the strong associations of psoriasis with psychosomatic and social complications in this patient group.

Within the framework of the study, the following research hypotheses were formulated:

1. The psychological state of patients with psoriasis and their preferred coping strategies in stressful situations influence their perception of quality of life and disease acceptance.
2. The level of disease acceptance correlates with the sense of quality of life in psoriasis patients.

## 2. Materials and Methods

### 2.1. Participants

The study was conducted amongst the patients of the Dermatology and Medical Cosmetics Clinic run by Prof. Wiaczesław Niczyporuk in Białystok and the Dermatology and Aesthetics Clinic run by Dr. Piotr Aleksiejczuk. The study included a group of 111 individuals with common psoriasis (46.8% women and 53.2% men). Respondents were divided into two age groups: 30–50 years old (53.2%) and over 50 years old (46.8%). The majority of respondents (82.9%) were residents of urban areas. In terms of occupational activity, physical workers (39.6%), intellectual workers (29.7%), and retirees (19.8%) predominated. The remaining respondents (10.8%) were pensioners, farmers, students, or individuals receiving social welfare benefits. As far as education is concerned, individuals with secondary education predominated (46.8%); one-third of the respondents (33.3%) had higher education, while the remaining respondents (19.8%) had primary or vocational education. Additionally, 55.9% of the respondents were married, while the rest were single (44.1% in total, including 17.1% divorced).

The inclusion criteria for the study were as follows: a diagnosis of common psoriasis for at least 0.5 years, the absence of other types of psoriasis, the absence of psychiatric disorders, and informed consent of the respondent to participate in the study. Potential respondents were excluded if their disease duration was less than 0.5 years, if they had been diagnosed with or had had other types of psoriasis coexisting, psychiatric disorders, or if they did not consent to participate in the study. Conducting the study was complicated by the onset of the COVID-19 pandemic. Initially, it was planned to collect a minimum of 200 complete questionnaires. Due to the epidemiological threat and restrictions, the authors aimed to collect no fewer than 100 fully completed surveys. A total of 200 survey forms were distributed, with some surveys not returned, some forms incompletely filled, and some qualified individuals opting out of participation while completing the survey. The response rate was 55.5% (*n* = 111), and the rejection rate was 44.5% (n = 89).

The respondents received paper questionnaires along with completion instructions, which they could complete independently either in the offices or at home. If needed, respondents could complete the questionnaire with the assistance of an interviewer (a member of the research team) in the office. Respondents who completed the questionnaire at home, after discussing it with the interviewer, also received an envelope with a pre-filled return address and postage stamped on it (to facilitate the return of the questionnaire). Additionally, the interviewer provided respondents with a phone number of a team member in case they had any questions during the completion of the questionnaire at home.

### 2.2. Measures

The study utilised a short, proprietary questionnaire and standardised scales, including the Dermatology Life Quality Index (DLQI) developed by Finlay and Khan [28], and adapted by Szepietowski et al., Polish researchers [36]; the Acceptance of Illness Scale (AIS) developed by Felton, Revenson and Hinrichsen, and adapted by the Polish researcher Juczyński [37]; and the Coping Inventory for Stressful Situations (CISS) developed by Endler and Parker, and adapted by Szczepaniak et al., Polish researchers [38].

#### 2.2.1. Proprietary Questionnaire

The proprietary questionnaire mainly covered questions regarding the socio-demographic characteristics of the respondents (gender, age, marital status, educational background, place of residence). Additionally, respondents were asked about the duration of psoriasis, their attitude towards the disease, and the location of psoriatic lesions.

#### 2.2.2. Dermatology Life Quality Index (DLQI)

The DLQI questionnaire is a standardised measure of the impact of skin conditions on the quality of life [28,36]. The scale assesses the patient’s condition over the past week, focusing particularly on disability and impaired functioning due to the skin disease. It assesses emotional effects of the disease to a lesser extent (one item out of ten asks about emotions related to the existence of the disease) [28,36]. The DLQI consists of 10 questions, with responses constructed on a 4-point Likert scale and assigned scores (very much—3 points, a lot—2 points, a little—1 point, not at all—0 points). Scores range from 0 to 30 points. A higher score on the DLQI indicates a more impaired quality of life for the patient since it measures the adverse impact of the disease on the quality of life [28,36].

#### 2.2.3. Acceptance of Illness Scale (AIS)

The acceptance of the disease was assessed using the standardised AIS questionnaire that consists of 8 statements related to a poor health status, limitations imposed by the disease, dependency on others, and reduced self-esteem. Respondents answer each question on a 5-point scale, indicating their current health status as follows: 1—‘strongly agree’ (indicating poor adaptation to the disease), 2—‘agree’, 3—‘unsure’, 4—‘disagree’, 5—‘strongly disagree’ (indicating acceptance of the disease) [37]. The respondent can score from 8 to 40 points. A higher score indicates better adaptation to the disease and its imposed limitations. In order to assess the level of acceptance, the score range is categorised into three intervals, thereby transforming the point scale into a three-level adjective scale. Scores from 8 to 18 indicate a lack of acceptance of the disease, scores from 19 to 29 indicate a moderate acceptance of the disease, and scores from 30 to 40 indicate a good acceptance of the disease [37].

#### 2.2.4. Coping Inventory for Stressful Situations (CISS)

The CISS is a questionnaire designed to assess coping strategies in stressful situations. It consists of 48 statements describing a respondent’s behaviour in stressful situations. Respondents use a 5-point scale to indicate how frequently they engage in each behaviour in challenging or stressful situations. A score of 1 point indicates the absence of any such behaviour, while a score of 5 points indicates very frequent engagement in that behaviour.

The results are grouped into three subscales, known as coping strategies: Task-oriented coping (TOC), in which the person focuses on tasks aimed at solving the problem; Emotion-oriented coping (EOC), in which the person focuses on themselves and their own experience, often experiencing anger or depression; and Avoidance-oriented coping (AOC), in which the person avoids thinking about the problem at hand. Scores for each subscale are summed according to a key, and respondents can score between 16 and 80 points in each subscale. A higher score in a particular subscale indicates that the respondent uses that coping style more frequently [38].

### 2.3. Procedure and Ethical Considerations

The research conformed with the Good Clinical Practice guidelines, and the procedures followed were in accordance with the Helsinki Declaration of 1975, as revised in 2000 (concerning the ethical principles for the medical community and forbidding release of the patient’s name and initials or the hospital evidence number). The study was reviewed and approved by the Bioethics Committee of the Medical University in Bialystok (resolution no APK.002.245.2020 of 25 June 2020).

### 2.4. Statistical Analysis

The obtained results were subjected to statistical analysis using Statistica 7 Data Miner C QC PL software (StatSoft Poland, Krakow, Poland). The synthetic description of the study group was elaborated upon using the method of percentage comparisons. The results were presented in the form of descriptive statistics in the compared groups (mean, standard deviation, median, lower and upper quartiles, and minimum and maximum values). The analysis of the relationship between two ordinal numerical variables was determined using the Spearman’s rank correlation coefficient (r) and performing a statistical test of the significance of the observed relationship. The regression analysis assessed the impact of independent factors (e.g., gender) upon the dependent variable with numerical characteristics. In all the analyses, a statistically significant level of *p* < 0.05 was adopted [39,40].

## 3. Results

### 3.1. Information on the Disease

The duration of the disease in the studied population varied and ranged from 0.5 years to over 50 years. Based on the quartile values of 4 and 20 years, it is plausible to conclude that the majority of individuals became ill several to several dozen years ago. The median for the study population was 12 years, the standard deviation was 13, and the mean was 14.9.

The emotional attitude towards the disease varied among the respondents. The predominant feelings included surprise about the disease (27%), considering it a terrible stigma (17.1%), feeling tired because of the disease (16.2%), indifference (14.4%), and seeing it as fate (12.6%). The remaining respondents did not specify their attitude towards the disease.

**Figure 1:**
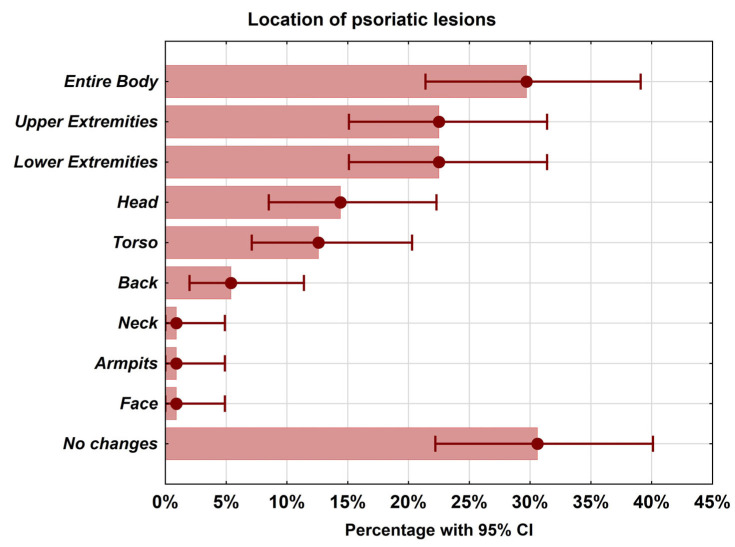
The location of psoriatic lesions. The total does not have to be 100% because respondents could indicate any number of response options.

The majority of the respondents declared that they had not fully come to terms with the disease (35.1%) or would never accept it (34.2%). It did not interfere with the lives of 18.9% of respondents, while 11.7% of respondents did not have a specific opinion. The majority of the respondents (29.7%) reported the presence of psoriatic lesions on their entire body, as well as on their upper and lower extremities (22.5% each) (Figure 1).

### 3.2. Distribution of Psychometric Measures

Among the respondents, relatively low values of the DLQI predominated (average of 10.8 points), indicating that the discomfort associated with the disease was not extremely burdensome for the majority of the surveyed individuals. The overall results for the studied group ranged from 0 to 28 points. The individuals with psoriasis included in this study primarily employed the Task-oriented coping strategy (TOC) in stressful situations, averaging around 54 points; followed by the Avoidance-oriented coping strategy (AOC), with an average of approximately 50 points; and least frequently, the Emotion-oriented coping strategy (EOC), with an average of about 46 points. The average degree of disease acceptance in the studied group was around 26 points, which was slightly above the average (Table 1).

### 3.3. Correlations between Measures Related to Reaction to the Disease and Coping Strategies in Stressful Situations

**Figure 2:**
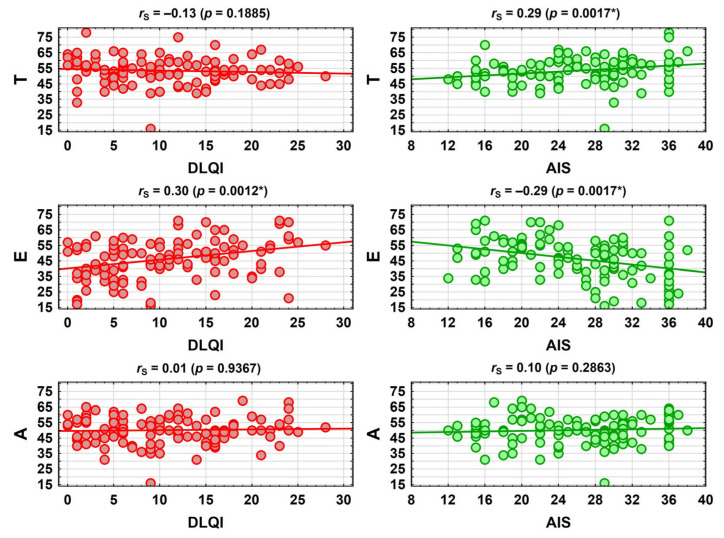
Correlations between the CISS, DLQI, and AIS measures. **Abbreviations:** AIS—Acceptance of Illness Scale; CISS—Coping Inventory for Stressful Situations; T—Task-oriented coping strategy; E—Emotion-oriented coping strategy; A—Avoidance-oriented coping strategy; DLQI—Dermatology Life Quality Index; *r*_S_—Spearman’s rank correlation coefficient; *p*—*p*-value; *—statistically significant correlations (*p* < 0.05).

Firstly, correlations between the CISS measures, the DLQI measures, and the AIS measures were analysed by means of the Spearman’s rank correlation coefficient. It was found that there was a statistically significant, small positive correlation between the Emotion-oriented coping strategy (EOC) and the Dermatology Life Quality Index (DLQI), as well as between the Task-oriented coping strategy (TOC) and disease acceptance (AIS). Additionally, a statistically significant, small negative correlation was observed between the Emotion-oriented coping strategy (EOC) and disease acceptance (AIS) (Figure 2).

### 3.4. Regression Models

In order to examine more closely how the AIS and DLQI measures were related to the CISS measures, a regression analysis was carried out. Separate models were constructed for the Task-oriented coping strategy (TOC) and the Emotion-oriented coping strategy (EOC) measures. The analysis also incorporated accompanying variables describing the demographic structure (gender, age, education) and the location of disease manifestations. Furthermore, throughout the model construction process, all possible second-degree interactions between accompanying variables and the AIS and DLQI were considered. An optimal model was sought in order to include only factors (or interactions between them) that were statistically significant, while maximising the model’s fit to the data. As a result, two models were obtained, and are presented in Table 2 and Table 3.

The variability of the Task-oriented coping strategy measure in the studied population was explained in approximately 15% of cases. The model included the AIS measure and gender interacting with the presence of lesions on the torso and head. As far as the influence of the AIS on the Task-oriented coping strategy is concerned, according to the correlation analysis results, an increase in disease acceptance correlated with a higher frequency of using the Task-oriented (rational) problem-solving style in stressful situations. The presence of psoriatic lesions on the torso led to a less rational approach in stressful situations (decrease in the use of the Task-oriented coping strategy) in women, but the opposite was observed in men. Conversely, the presence of psoriatic lesions on the head had a stimulating effect on the use of the Task-oriented coping strategy in women, while the opposite effect was seen in men (Table 2).

**Figure 3:**
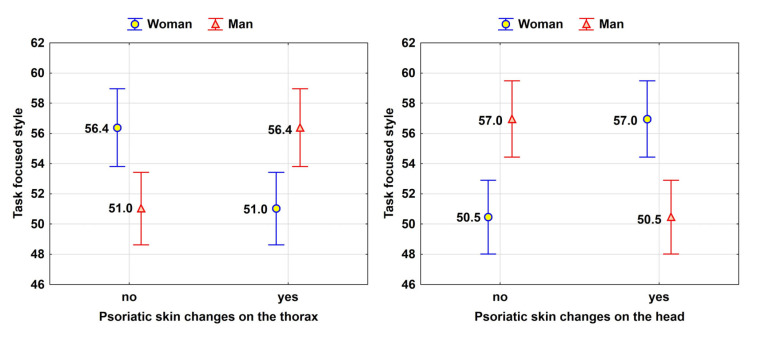
TOC, gender, and location of skin lesions.

The Figure displays the interaction effects in the regression model (expected TOC values estimated for respective groups based on the regression model) (Figure 3).

In the regression model for the Emotion-oriented coping strategy (EOC), three factors were identified: age (the group aged 31–50 compared to others), DLQI, and AIS. The higher disease acceptance correlated with less frequent emotional approaches in stressful situations, and the higher quality of life (lower DLQI) correlated with lower EOC values. Therefore, it is plausible to conclude that individuals experiencing fewer problems with psoriasis tend to be less emotional in stressful situations, whereas individuals aged 31–50 exhibit more emotional responses in stressful situations compared to younger or older individuals (Table 3).

## 4. Discussion

People suffering from psoriasis struggle with a negative self-image, and the condition evokes various emotions in them. In our study, respondents exhibited diverse emotional reactions towards the disease. The predominant feelings among them included surprise about the disease (27%), considering it a terrible stigma (17.1%), feeling tired of the disease (16.2%), indifference (14.4%), and perceiving it as fate (12.6%). In the study conducted by Linder et al. [41] psoriasis most commonly evoked anger and irritation due to the inconveniences associated with the disease (about 50% of the patients). Additionally, 38% of patients were unable to describe their emotional state regarding the condition.

It should be emphasised that psoriasis, due to the location of symptoms primarily on the skin and thus being visible, not only disrupts one’s self-image but also adversely impacts the quality of life (QOL). The QOL of psoriasis patients may decrease with an exacerbated severity and extent of skin lesions, dysfunctionality resulting from the disease, depending on the psychological makeup of the patients. In our study, the majority of patients had relatively low DLQI scores (average of 10.8 points), indicating that the impact of the disease-related symptoms was not extremely burdensome for most respondents. The overall results for the study group ranged from 0 to 28 points on the DLQI scale. In the study conducted by Sendrasoa et al. [42] the average DLQI score was higher at 13.8 points. Symptoms, feelings, and psychological impact were the most affected dimensions. The quality of life was poorer among young, single patients with average levels of education. Although patients with a disease duration of more than 5 years had a higher DLQI score compared to others, that difference was not statistically significant. Furthermore, the clinical presentation of psoriasis did not significantly influence the quality of life of patients [42]. In the study conducted by Tang et al. [43] 48% of patients reported that their disease had a very large or large impact on their quality of life (DLQI, 11–30 points), while only 13% of patients reported no impact of psoriasis on their quality of life (DLQI, 0–1 points). As reported by Šmejkalová et al. [44] in their study of 51 patients with severe plaque psoriasis, the average PDI (Psoriasis Disability Index) score upon admission was 9.02 and did not change significantly one month after discharge, despite effective treatment. The authors highlight that the prolonged duration of the disease and adaptation to living with psoriasis likely influenced the results they obtained.

Given the above, it appears crucial for patients to achieve acceptance of their health condition and consequently come to terms with the disease and its consequences or symptoms. Disease acceptance is an individual and subjective aspect that depends on various factors, including the duration of the disease, the severity of its symptoms and impairments, the psychological makeup of the patient, the support or lack thereof from family and others in the environment, and many other factors.

In our study group, the average level of disease acceptance, measured by means of the AIS (Acceptance of Illness Scale), was approximately 26 points, indicating a moderate level of acceptance of psoriasis. Furthermore, higher levels of disease acceptance correlated with less frequent emotional responses in stressful situations and a better quality of life among the participants. It is noteworthy that the location of skin lesions remained associated with the type of stress response observed in both women and men. It can therefore be assumed that people with better acceptance of the disease and a higher quality of life were characterized by having better strategies for solving problems or stressful situations and, as a result, experienced a smaller expansion of disease lesions. A similar study result was reported by Soliman [45], in which patients experiencing a higher impairment in their quality of life reported lower levels of disease acceptance and a greater need for education and support. However, Kostyła et al. [17] confirmed the existence of a relationship between the degree of acceptance of skin disease and the severity of psychopathological symptoms in patients with psoriasis (negative correlations). Demographic factors did not have any impact upon the disease acceptance within the framework of the study conducted by Zalewska et al. [46], but based on a multiple regression analysis, the authors demonstrated that higher levels of optimism, lower beliefs in the influence of others on health, and less frequent use of emotion-oriented coping strategies, along with more severe disease in terms of the PASI scores, correlated with higher disease acceptance in patients with psoriasis [46]. In our own study, correlations were examined between the CISS measures, the DLQI measures, and the AIS measures. Significant correlations were found between the CISS-EOC and DLQI, and between the AIS and CISS-TOC as well as the CISS-EOC. The influence of the AIS on the Task-oriented coping strategy (TOC), according to the correlation analysis results, indicates that as the disease acceptance increases, the frequency of using the Task-oriented (rational) problem-solving style in stressful situations also increases. The presence of psoriatic lesions on the torso led to less rational attitudes in stressful situations (decrease in the use of the TOC) among women, but the opposite result was observed in men. In summary, higher disease acceptance among individuals with psoriasis in our study was associated with less frequent emotional approaches in stressful situations. It can be assumed that the location of lesions on the trunk of women was more traumatic for them. Thus, this caused a situation that was difficult to reconcile due to the negative modification of the image in which unfavorable changes were noticed by people around them. The subsequent situations significantly influenced women’s reactions, which became less constructive and more emotional. Furthermore, a higher quality of life (lower DLQI) was associated with lower EOC values. Thus, individuals experiencing fewer problems with psoriasis tended to be less emotional in stressful situations, while individuals aged 31–50 years tended to exhibit more emotional responses compared to younger or older age groups. Hughes et al. [47], after having analysed the data from 231 psoriasis patients, found both men and women to have faced daily practical, social, and emotional challenges and coped with them in various ways. Higher levels of anxiety about their appearance, influenced by more severe skin lesions and a younger age, were evident. Both men and women seemed equally emotionally affected by living with the dermatosis, with a significant gender difference being that women more frequently engaged in an avoidance-oriented approach related to coping strategies compared to men [47].

In our own study, we have aimed to determine coping strategies in stressful situations caused by psoriasis by means of the CISS scale. Psoriasis patients in our study predominantly employed the Task-oriented coping strategy (TOC) when faced with stressful situations, with an average score of approximately 54 points. The Avoidance-oriented coping strategy (AOC) was the next most common coping strategy, with an average score of around 50 points, followed by the Emotion-oriented coping strategy (EOC), which was the least frequently used, with an average score of about 46 points. Noormohammadpour et al. [48] examined the CISS scores based on the location of skin lesions. The lowest coping score was reported by individuals with lesions on their hands (50.5 points), while the highest was observed in those with lesions on their genital organs (60.4 points). We have found a significant correlation between psychological sensitivity and the perception of disease severity and between psychological sensitivity and the coping strategy score for dealing with the disease [48]. Karabi et al. [49] chose to assess stress severity using the Perceived Stress Scale (PSS) in two groups: individuals with psoriasis (195 subjects) and a control group of healthy individuals (114 subjects). The results obtained by the authors were significantly higher in the psoriasis group compared to the control group (25.14 ± 8.67 vs. 23.0 ± 6.93). Subscale results of the PSS for perceived self-efficacy (PSE) were significantly higher in psoriasis patients compared to the control group. Additionally, the data obtained from the PSE were positively correlated with the Physician Global Assessment (PGA) scores. Therefore, this study also confirms the role of stress in psoriasis, and effective stress management may impact therapy outcomes. This aspect is also highlighted by Seville [50], who described a study involving 132 patients observed over a 3-year period after remission, in which the recurrence of psoriasis symptoms ranged from 2 days to 1 month after the occurrence of stress. In the study, 39% of participants reported to have experienced specific stress within a month before their first disease flare-up, and importantly, the prognosis in this group was better.

Living with a skin condition poses daily multidimensional challenges—psychological, social, emotional, and practical—for both men and women. It highlights the necessity for routine consideration of these issues in the care of patients with psoriasis. Beyond practical measures aimed at managing skin lesions, psychosocial interventions are crucial. They equip patients with tools to cope with the psychological and emotional consequences of dermatoses and help shape social environments so that individuals with dermatological conditions do not feel stigmatised in their interactions with others.

### Limitations

The major limitation of our study is the relatively small sample size of 111 patients with psoriasis. Originally, we aimed for a larger sample size, but recruitment was hindered by the outbreak of the COVID-19 pandemic. Other limitations include the fact that the patients were recruited from a single region, specifically the Podlaskie Voivodeship. Additionally, we noted a lack of specific detailed data on the location of psoriatic lesions, especially in sensitive areas such as genital organs, face, and nails, which could influence patients’ psychological reactions. It is also important to acknowledge that data obtained using psychometric scales depend on the individual circumstances of the study participants, and their results may vary depending on the timing of the study. Another limitation of the study is the lack of analysis in the context of the severity of psoriatic lesions, which was not covered by this study.

## 5. Conclusions

As the disease acceptance (AIS) increased, the frequency of using the Task-oriented coping strategy (TOC) in stressful situations also increased. Psoriatic lesions on the torso led to less frequent use of the Task-oriented coping strategy (TOC) in stressful situations among women, but the opposite effect was observed in men. Conversely, psoriatic lesions on the head stimulated the use of the Task-oriented coping strategy (TOC) among women but had the opposite effect in men. A higher disease acceptance (AIS) was associated with less frequent use of the Emotion-oriented coping strategy (EOC) in stressful situations, while a higher quality of life (DLQI) was associated with lower values of the Emotion-oriented coping strategy (EOC). Individuals experiencing fewer problems with psoriasis tended to be less emotional in stressful situations. However, individuals aged 31–50 years tended to exhibit more emotional responses compared to younger or older age groups.

## Figures and Tables

**Table 1:** DLQI, CISS & AIS values for the whole study population.

Scale (pts)		M	Me	SD	Q_25_	Q_75_	Min	Max
DLQI		10.8	10	7.3	5	16	0	28
CISS	T	53.6	54	8.7	49	59	16	78
	E	46.2	48	12.7	38	55	16	71
	A	50.1	50	8.7	45	56	16	69
AIS		26.1	27	6.9	20	31	12	38

**Abbreviations:** AIS—Acceptance of Illness Scale; CISS—Coping Inventory for Stressful Situations; T—Task-oriented coping strategy; E—Emotion-oriented coping strategy; A—Avoidance-oriented coping strategy; DLQI—Dermatology Life Quality Index; M—mean; Max—maximum; Me—median; Min—minimum; SD—standard deviation; Q_25_—lower quartile; Q_75_—upper quartile.

**Table 2:** Acceptance of the disease and the Task-oriented coping strategy when dealing with stress vary with gender and the location of skin lesions.

Independent Factors	Task-Oriented Coping Strategy *R*^2^ = 15.2%, *F* = 6.4, *p* = 0.0005 *		
	*B* (95% CI)	*p*	*ß*
AIS	0.301 (0.074; 0.528)	0.0096 *	0.24
Gender (male vs. female) × lesions on torso (yes vs. no)	5.768 (1.444; 9.129)	0.0078 *	0.31
Gender (male vs. female) × lesions on head (yes vs. no)	−6.504 (−10.416; −2.592)	0.0013 *	−0.38

**Abbreviations:** AIS—Acceptance of Illness Scale; T—Task-oriented coping strategy; *R*^2^—coefficient of determination; *F*—test statistics; *p*—*p*-value for significance of the whole model; *B*—regression coefficient with 95% CI.; *p*—*p*-value for significance of each regression coefficient; *ß*—standardised regression coefficient; *—statistically significant.

**Table 3:** Emotion-oriented coping strategy and respondents’ age and DLQI and AISS values.

Independent Factors	Emotion-Oriented Coping Strategy *R*^2^ = 16.5%, *F* = 7.1, *p* = 0.0002 *		
	*B* (95% CI)	*p*	*ß*
Age (31–50 vs. others)	4.170 (−0.419; 8.760)	0.0745	0.16
DLQI	0.310 (−0.054; 0.675)	0.0945	0.18
AIS	−0.429 (−0.814; −0.044)	0.0294 *	−0.23

**Abbreviations:** AIS—Acceptance of Illness Scale; DLQI—Dermatology Life Quality Index; E—Emotion-oriented coping strategy; *R*^2^—coefficient of determination; *F*—test statistics; *p*—*p*-value for significance of the whole model; *B*—regression coefficient with 95% CI.; *p*—*p*-value for significance of each regression coefficient; *ß*—standardised regression coefficient; *—statistically significant.

## Data Availability

Data are available upon reasonable request.
